# Population pharmacokinetic analysis of doripenem for Japanese patients in intensive care unit

**DOI:** 10.1038/s41598-020-79076-6

**Published:** 2020-12-17

**Authors:** Ko Nonoshita, Yosuke Suzuki, Ryota Tanaka, Tetsuya Kaneko, Yoshifumi Ohchi, Yuhki Sato, Norihisa Yasuda, Koji Goto, Takaaki Kitano, Hiroki Itoh

**Affiliations:** 1 Department of Clinical Pharmacy, Oita University Hospital, Hasama-machi, Oita, Japan; 2 Department of Anesthesiology and Intensive Care, Faculty of Medicine, Oita University, Hasama-machi, Oita, Japan

**Keywords:** Continuous renal replacement therapy, Antimicrobial therapy

## Abstract

We aimed to construct a novel population pharmacokinetics (PPK) model of doripenem (DRPM) for Japanese patients in intensive care unit, incorporating the clearance of DRPM by continuous renal replacement therapy (CRRT). Twenty-one patients treated with DRPM (0.25 or 0.5 g) by intravenous infusion over 1 h were included in the study. Nine of the 21 patients were receiving CRRT. Plasma samples were obtained before and 1, 2, 4, 6 and 8 h after the first DRPM administration. PPK analysis was conducted by nonlinear mixed effects modeling using a two-compartment model. Total clearance (CL_total_) in the model was divided into CRRT clearance (CL_CRRT_) and body clearance (CL_body_). The final model was: CL_total_ (L h^−1^) = CL_body(non-CRRT)_ = 3.65 × (Ccr/62.25)^0.64^ in the absence of CRRT, or = CL_body(CRRT)_ + CL_CRRT_ = 2.49 × (Ccr/52.75)^0.42^ + CL_CRRT_ in the presence of CRRT; CL_CRRT_ = Q_E_ × 0.919 (0.919 represents non-protein binding rate of DRPM); V_1_ (L) = 10.04; V_2_ (L) = 8.13; and Q (L h^−1^) = 3.53. Using this model, CL_total_ was lower and the distribution volumes (V_1_ and V_2_) tended to be higher compared to previous reports. Also, Ccr was selected as a significant covariate for CL_body_. Furthermore, the contribution rate of CL_CRRT_ to CL_total_ was 30–40%, suggesting the importance of drug removal by CRRT. The population analysis model used in this study is a useful tool for planning DRPM regimen and administration. Our novel model may contribute greatly to proper use of DRPM in patients requiring intensive care.

## Introduction

Population pharmacokinetic (PPK) analysis provides PPK parameters consisting of average and variance for a population using the nonlinear mixed effect model (NONMEM)^1^ that fits all the drug plasma concentrations of multiple patients^2^. Individual pharmacokinetic (PK) parameters such as clearance (CL) and distribution volume (V_d_) are obtained by Bayesian method using PPK parameters^3^.

Doripenem (DRPM) is a carbapenem antibacterial agent that has a broad antibacterial spectrum against Gram-positive bacteria, Gram-negative bacteria, and anaerobic bacteria^4^. Since DRPM shows a time-dependent effect based on the pharmacokinetic and pharmacodynamics (PK/PD) theory, bactericidal effect is greater when the blood concentration is maintained above the minimum inhibitory concentration (MIC) for 40% of the time or more^5^.

Several PPK analysis models for DRPM have been reported. The PPK model reported by Bhavnani et al.^6^ was constructed using an intravenous 2-compartment model based on plasma DRPM concentrations obtained in a phase 1 DRPM clinical trial. The PK parameters they reported included total CL (CL_total_), inter-compartmental clearance (Q), distribution volume of central compartment, and distribution volume of peripheral compartment. In addition, Lee et al.^7^ succeeded to build a good PPK model for patients with sepsis by integrating creatinine clearance (Ccr) as a covariate in CL_total_ using an intravenous 1-compartment model. In the PPK analysis reported by Nandy et al.^8^, covariates such as Ccr, body weight (BW), age and race were incorporated using an intravenous 2-compartment model for a wide range of subjects in phases 1–3 trials. They reported differences in DRPM clearance depending on race. Reports suggested that some degree of uniformity of the population including race may be necessary because racial variation may affect the PK of drugs such as distribution, metabolism, and elimination^9,10^. Matsuo et al.^11^ reported a PPK model of DRPM targeting Japanese subjects by incorporating Ccr and age as covariates in CL_total_, using an intravenous 2-compartment model in phase 1 healthy subjects.

A retrospective cohort study conducted by Kumar et al.^12^ showed that the death rate increased by 7.6% when administration of antibiotics was delayed by 1 h. Other reports^13–15^ also suggested that the risk of death in septic shock patients decreased if antibiotics were administered within 1 h. There is a relationship between the time to start of antibiotic administration and death^16^, and international guideline has recommended to administer antibiotics within 1 h after diagnosis^17^. Carbapenem antibiotics are used empirically in the intensive care unit (ICU) for complex and severe infections. Water-soluble drugs with low molecular weight and low protein binding rate, such as carbapenems, are likely to show increased V_d_ and higher CL in critically ill patients^18,19^. In critically ill patients in ICU, CRRT is often performed due to declined kidney function such as acute kidney injury and for removal of inflammatory cytokines. Therefore, clearance of DRPM from the body by CRRT should be considered in patients undergoing CRRT. Using an intravenous 2-compartment model that considers the clearance of DRPM by CRRT, Roberts et al.^20^ constructed a PPK model without incorporating covariates for critically ill patients undergoing CRRT. They reported that it was necessary to consider the removal of DRPM by CRRT, since clearance by CRRT (CL_CRRT_) contributed to 30–37% of CL_total_. In addition, Monte Carlo simulation using the PK parameters obtained suggested that the change in distribution volume substantially affected the time above MIC.

In this study, we aimed to construct a novel PPK model incorporating optimal covariates including CRRT in each PK parameter for Japanese ICU patients.

## Material and methods

### Patients

This study was conducted in accordance with the Declaration of Helsinki. The study was started after obtaining approval from the Ethics Committee in Oita University (Approval No. 613). The subjects in this study consisted of 21 inpatients (a total of 97 samples) in ICU treated with DRPM, who gave written informed consent obtained from either the patients or their legally authorized representatives. Nine of the 21 patients were receiving CRRT. Patients who were administered other carbapenem antibacterial agents before administration of DRPM were excluded.

CRRT was conducted by continuous hemodiafiltration. The hemofilter was a cellulose triacetate membrane. The conditions for CRRT were: blood flow rate (Q_B_) = 80–100 mL min^−1^; dialysate flow rate (Q_D_) = 0.3–0.9 L h^−1^; replacement fluid flow rate (Q_S_) = 0.3–0.9 L h^−1^; and filtrate flow rate (Q_E_) = 0.6–1.8 L h^−1^.

DRPM at a dose of 0.25 or 0.5 g was given by intravenous infusion over 1 h. At the first administration, plasma samples were obtained from blood sampling before DRPM administration and at 1, 2, 4, 6 and 8 h after the start of infusion. Plasma DRPM concentrations were measured by high performance liquid chromatography (HPLC) according to the procedures we reported previously^21^. In brief, plasma samples were pre-treated by a solid-phase extraction method. The HPLC system (Waters 2695) was used with a Shiseido Capcell Pak C18 MGII column (5 μm, 250 mm × 4.6 mm; Shiseido Co., Tokyo, Japan) and ultraviolet absorbance detection (Waters 2489 UV/Vis). Separation of DRPM and internal standard was satisfactory, and was free of interfering peaks from the plasma matrix. The limit of quantification (LOQ) for the DRPM assay was 0.5 μg mL^−1^, and the calibration curve was linear from 0.5 to 100 μg mL^−1^ (*r*^2^ = 0.999). Only one sample had a concentration below the LOQ.

### Population pharmacokinetics

**Figure 1:**
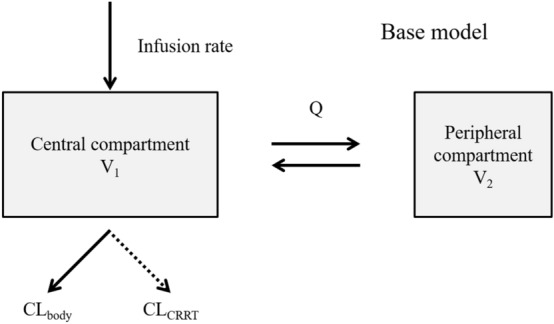
Base model for this study. *V*_*1*_ distribution volume of central compartment, *V*_*2*_ distribution volume of peripheral compartment, *Q* distribution clearance between the central and peripheral compartments, *CL*_*body*_ clearance from body, *CL*_*CRRT*_ clearance by continuous renal replacement therapy.

Analysis of population pharmacokinetics was conducted using nonlinear mixed effects modeling (NONMEM) version 7.3.0^22^. We selected the compartment model using the DRPM plasma concentration‒time curve with log-transform, by comparing the 1-compartment and 2-compartment models using objective function value (OFV) and the Akaike criteria (AIC)^23^. Since a conventional 2-compartment model would not include the route of DRPM removal by CRRT, we modified the conventional model to include CRRT clearance. Thus, total clearance in the model was divided into CRRT CL (CL_CRRT_) and body CL (CL_body_) using the ADVAN6 subroutines from the NONMEM library. We initially regarded this as the base model (Fig. 1). For each PPK parameter, inter-individual variability was evaluated by the exponential error model, and residual variability was evaluated using the additive error model. Clinically plausible covariates such as Ccr obtained by the Cockcroft–Gault equation^24^, BW, and albumin (Alb) were screened as PK parameters of DRPM. Screening of covariates was performed by addition of the candidate covariate to the base model. An eligible covariate should have a correlation with one PK parameter (correlation coefficient *r* > 0.6), and furthermore no correlation with other covariates; that is, no multiple collinearity. After addition of the covariate, a reduction in OFV of more than 2.71 for one degree of freedom was considered a statistically significant improvement (*p* < 0.10) based on the χ^2^ test. After selection of the covariates by the above forward addition step, the model was refined by backward elimination step. A covariate was included in the model when the significance level *p* < 0.05 (a reduction in the OFV of more than 3.84 for one degree of freedom based on the χ^2^ test) was obtained. This analysis was calculated by the first-order conditional estimation with interaction method.

### Model evaluation

Validity of our model was examined using visual predictive check and goodness-of-fit plots^25^. Also, evaluation of reliability and stability of the final model was performed by the bootstrap method. A thousand bootstrap data sets were reconstructed by resampling the subjects from the original data set. The average and standard deviation of parameter estimates obtained from the bootstrap were compared to the estimates of parameters for the final model and standard error obtained from the original data set.

## Results

### Patients

Patient demographics and relevant clinical data are summarized in Table 1. Among 21 patients, 18 patients were males and three were females. The mean ± standard deviation of Ccr was 68.0 ± 33.4 (mL min^−1^). Nine patients were undergoing CRRT during DRPM treatment, eight of whom had renal indications for CRRT.

**Table 1:** Demographics and relevant clinical data of all patients, those who underwent continuous renal replacement therapy (CRRT) and those who did not undergo CRRT.

	All patients	Non-CRRT	CRRT
No. of patients	21	12	9
Males/females	18/3	11/1	7/2
Age (year)	61.8 ± 18.9	63.8 ± 17.5	59.1 ± 20.3
Height (cm)	164.2 ± 9.1	166.1 ± 5.37	161.7 ± 12.1
Body weight (kg)	61.5 ± 13.6	62.1 ± 6.1	60.6 ± 19.5
Ccr (mL min^−1^)	68.0 ± 33.4	76.0 ± 35.8	57.3 ± 26.5
APACHE II score	17.6 ± 6.7	15.8 ± 4.9	20.0 ± 7.8
SOFA score	7.5 ± 2.6	6.6 ± 1.8	8.8 ± 3.0
Dose (500 mg/250 mg)	20/1	11/1	9/0

Data are expressed as number or mean ± S.D. Ccr, creatinine clearance; CRRT, continuous renal replacement therapy; APACHE II score, acute physiology and chronic health evaluation score II; SOFA score, sequential organ failure assessment score.

### Population pharmacokinetics

**Figure 2:**
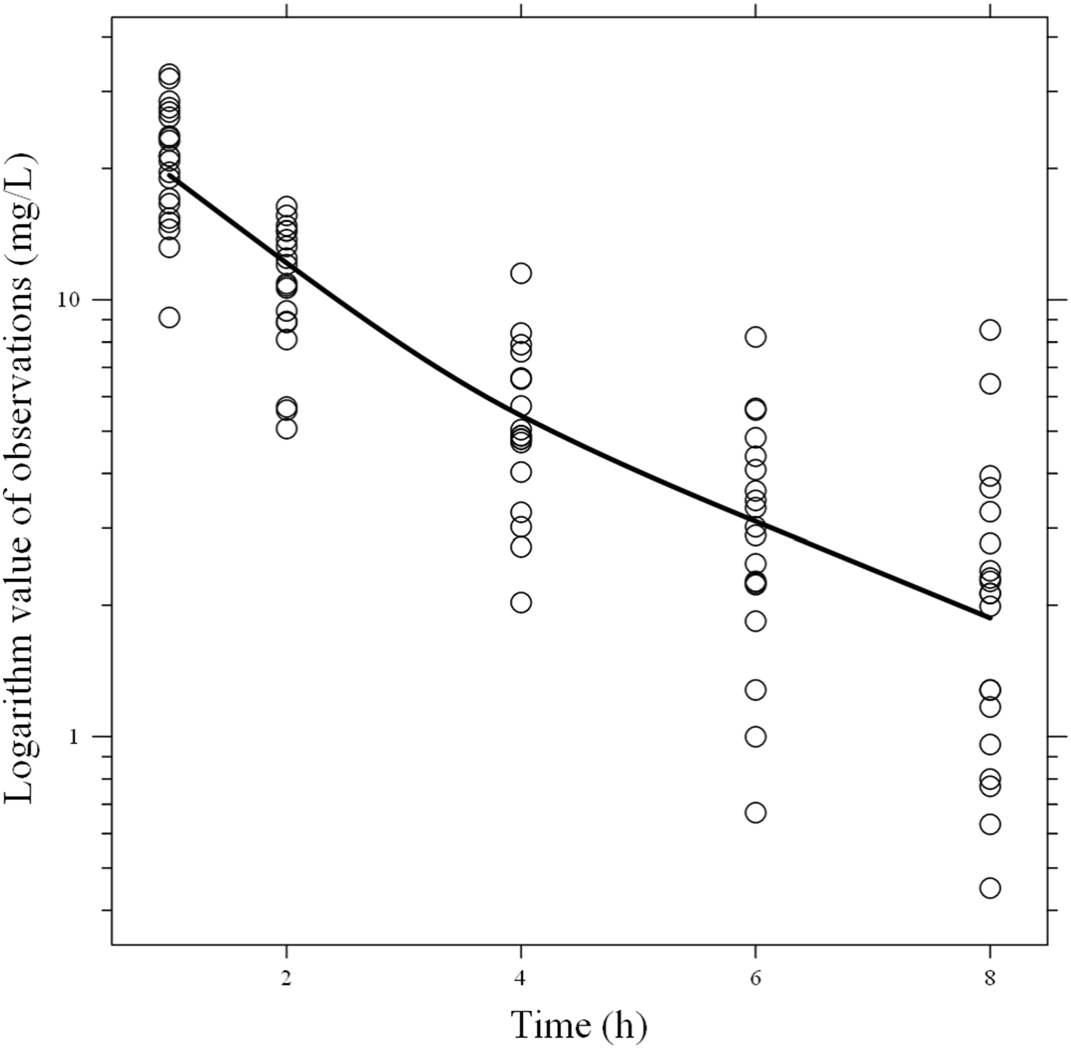
Semi-logarithm plot of doripenem plasma concentrations. The solid line shows the smooth fitting for doripenem plasma concentrations.

As shown in Fig. 2, a semi-logarithm plot of DRPM plasma concentration versus time showed two phases (distribution phase and elimination phase). Comparisons of 1-compartment and 2-compartment models using OFV and AIC confirmed that both the OFV and AIC of the 2-compartment model were significantly smaller than those of the 1-compartment model (ΔOFV = 89.8, *p* < 0.05; ΔAIC = 36.3, *p* < 0.05). Based on this finding, we adopted the 2-compartment model in this study. In addition, comparison with the conventional 2-compartment model without including CRRT clearance confirmed that OFV decreased significantly in our modified 2-compartment model (ΔOFV = 29.8, *p* < 0.001). Thus, this model that considers removal of DRPM by CRRT was used as the base model in this study.

After covariate selection by the forward addition step, models #8 was selected as the full model (Table 2). The full model incorporated Ccr in CL_body_, Alb in CL_CRRT_, and BW in V_1_. Ccr and BW have been selected as covariates in previous reports^7,8,11,26^, and Alb has been reported to be removed by continuous hemofiltration using a cellulose triacetate membrane^27^. Next, at the backward elimination step, when Ccr was excluded from the full model (model #8), OFV increased significantly (Table 2).

**Table 2:** Forward addition step and backward elimination step.

Model No	Model	OFV	ΔOFV	*p* value*
	Null model	230.06		
#0	Base model	200.23	29.83	< 0.05
**Forward addition**				
#1	#0 + Ccr in CL_body_	189.52	10.71	< 0.01
#2	#0 + Alb in V_1_	199.52	0.71	0.40
#3	#0 + Alb in V_2_	205.76	− 5.53	N/A
#4	#0 + Alb in CL_CRRT_	196.78	3.45	0.06
#5	#0 + BW in CL_body_	193.17	7.06	< 0.01
#6	#0 + BW in V_1_	197.22	3.01	0.08
#7	#0 + BW in V_2_	200.05	0.18	0.67
**Full model**				
#8	#0 + Ccr in CL_body_ and Alb in CL_CRRT_ and BW in V_1_	187.43	12.80	0.01
**Backward elimination**				
1	#8 – Ccr in CL_body_	195.48	8.05	< 0.02
2	#8 − Alb in CL_CRRT_	188.35	0.92	0.34
3	#8 − BW in V_1_	188.34	0.91	0.34

*OFV* objective function value, *ΔOFV* distribution of OFV between models, *Null model* normal 2-compartment model, *Base model* 2-compartment model considering clearance by continuous renal replacement therapy, *CL*_*body*_ clearance from body, *CL*_*CRRT*_ clearance by continuous renal replacement therapy, *V*_*1*_ distribution volume of central compartment, *V*_*2*_ distribution volume of peripheral compartment, *Ccr* creatinine clearance, *Alb* serum albumin, *BW* body weight.

*For one degree of freedom, ΔOFV below 2.71 or 3.84 was regarded as significant (*p* < 0.10 in forward addition, *p* < 0.05 in backward elimination).

**Figure 3:**
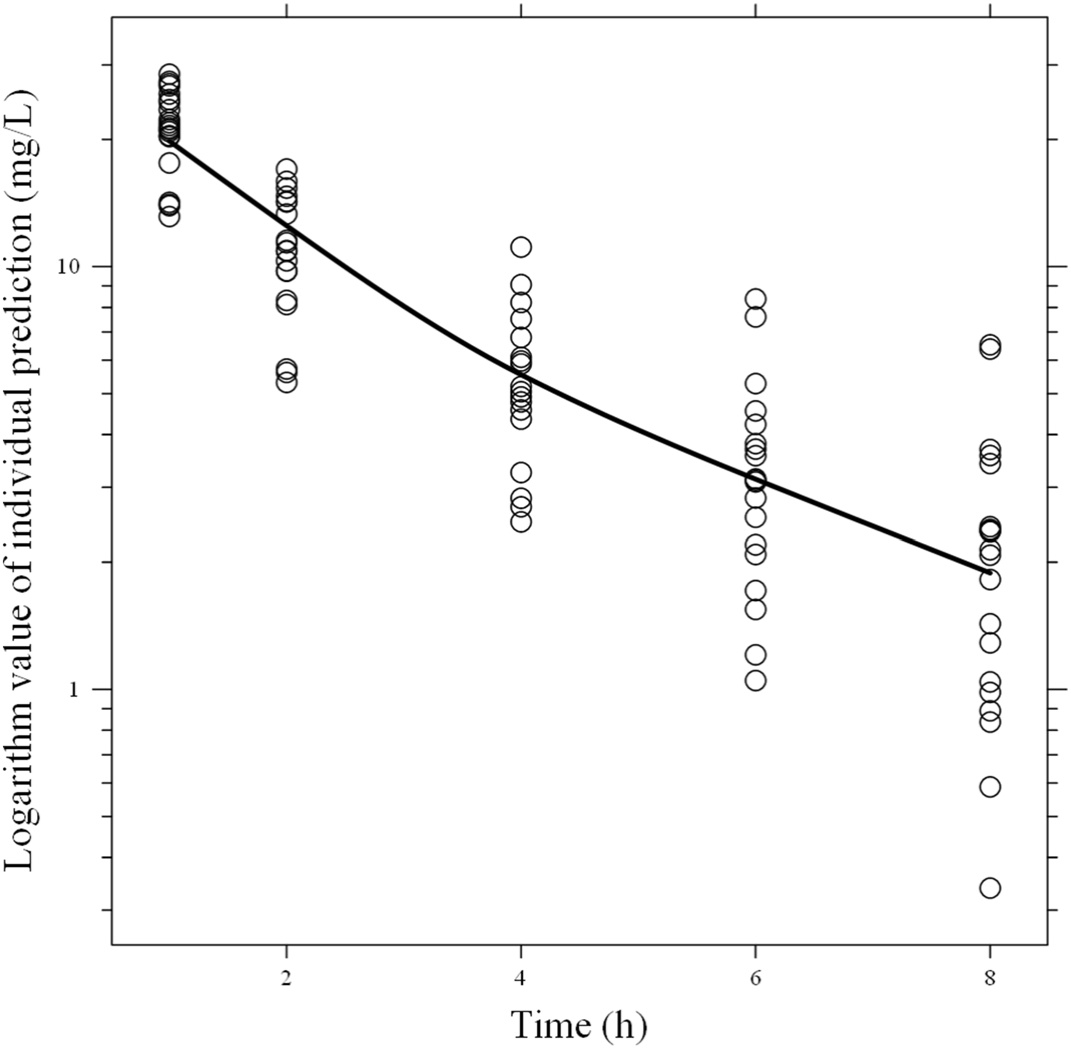
Semi-logarithm plot of individual prediction versus time. The solid line shows the smooth fitting for individual predictions.

The final model was: CL_total_ (L h^−1^) = CL_body(non-CRRT)_ = 3.65 × (Ccr/62.25)^0.64^ in the absence of CRRT, or = CL_body(CRRT)_ + CL_CRRT_ = 2.49 × (Ccr/52.75)^0.42^ + CL_CRRT_ in the presence of CRRT; CL_CRRT_ = Q_E_ × 0.919 (0.919 represents the non-protein binding rate of DRPM)^28^; V_1_ (L) = 10.04; V_2_ (L) = 8.13; and Q (L h^−1^) = 3.53. Inter-individual variability of CL_body(CRRT)_, CL_body(non-CRRT)_, V_1_, V_2_, and Q were 7.3%, 22.2%, 13.2%, 24.2%, and 12.7%, respectively; and residual variability was 36.5%. The η shrinkages^29^ of CL_body(CRRT)_, CL_body(non-CRRT)_, V_1_, V_2_, and Q were 36.4%, 33.6%, 56.1%, 11.8%, and 56.6%; and ε shrinkage was 23.9%. Figure 3 presents the plot of individual prediction versus time.

### Model evaluation

**Figure 4:**
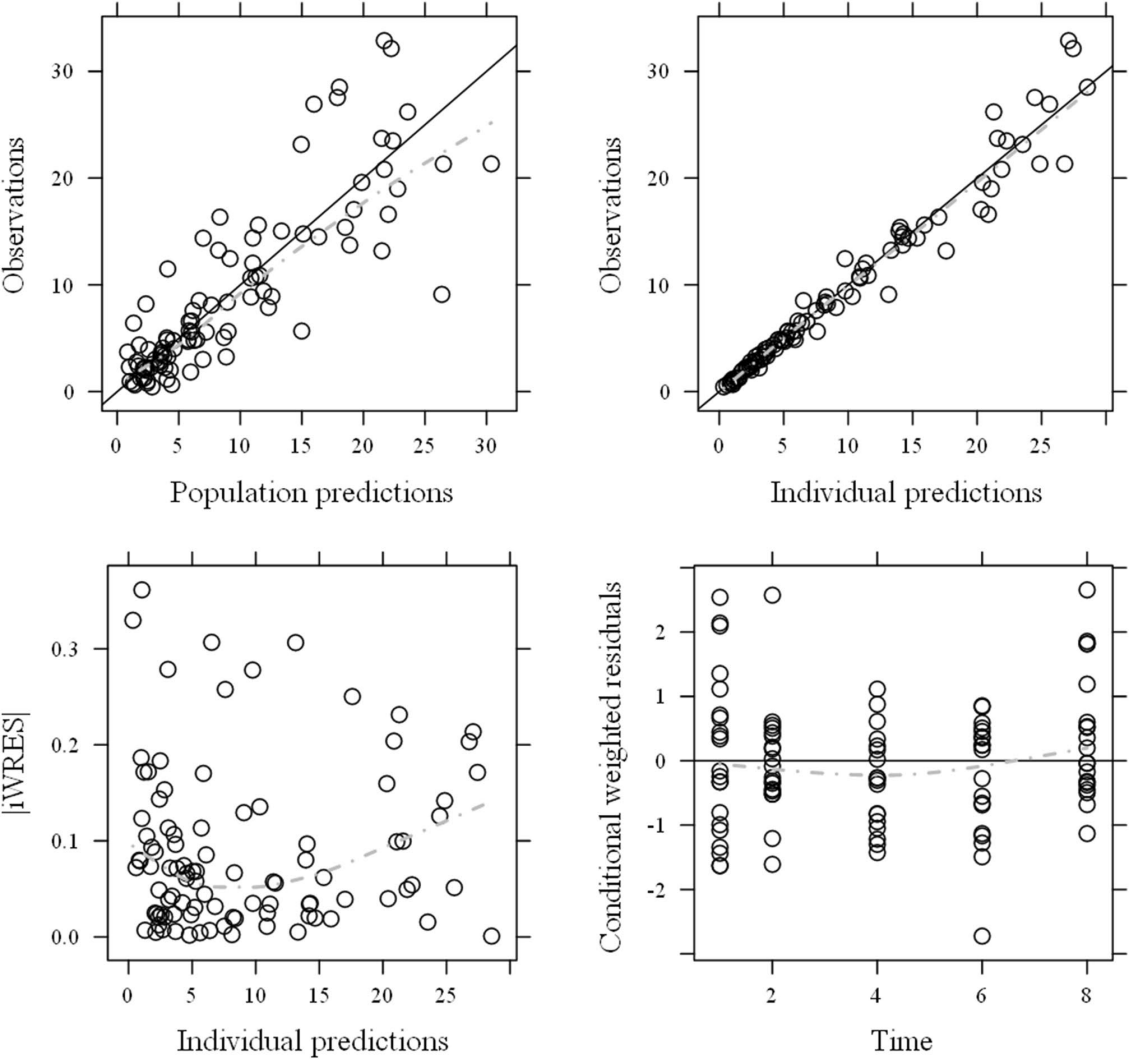
Goodness-of-fit plots for the final model; population prediction (PRED) versus observation (upper left), Bayesian-estimated individual prediction (IPRED) versus observation (upper right), individual weighted residual versus IPRED (lower left), and conditional weighted residual versus time (lower right). The open circles show observations. The solid black line shows the line of identity. The dotted and dashed gray line shows smooth fitting for observations. |iWRES|, absolute individual weighted residuals.

**Figure 5:**
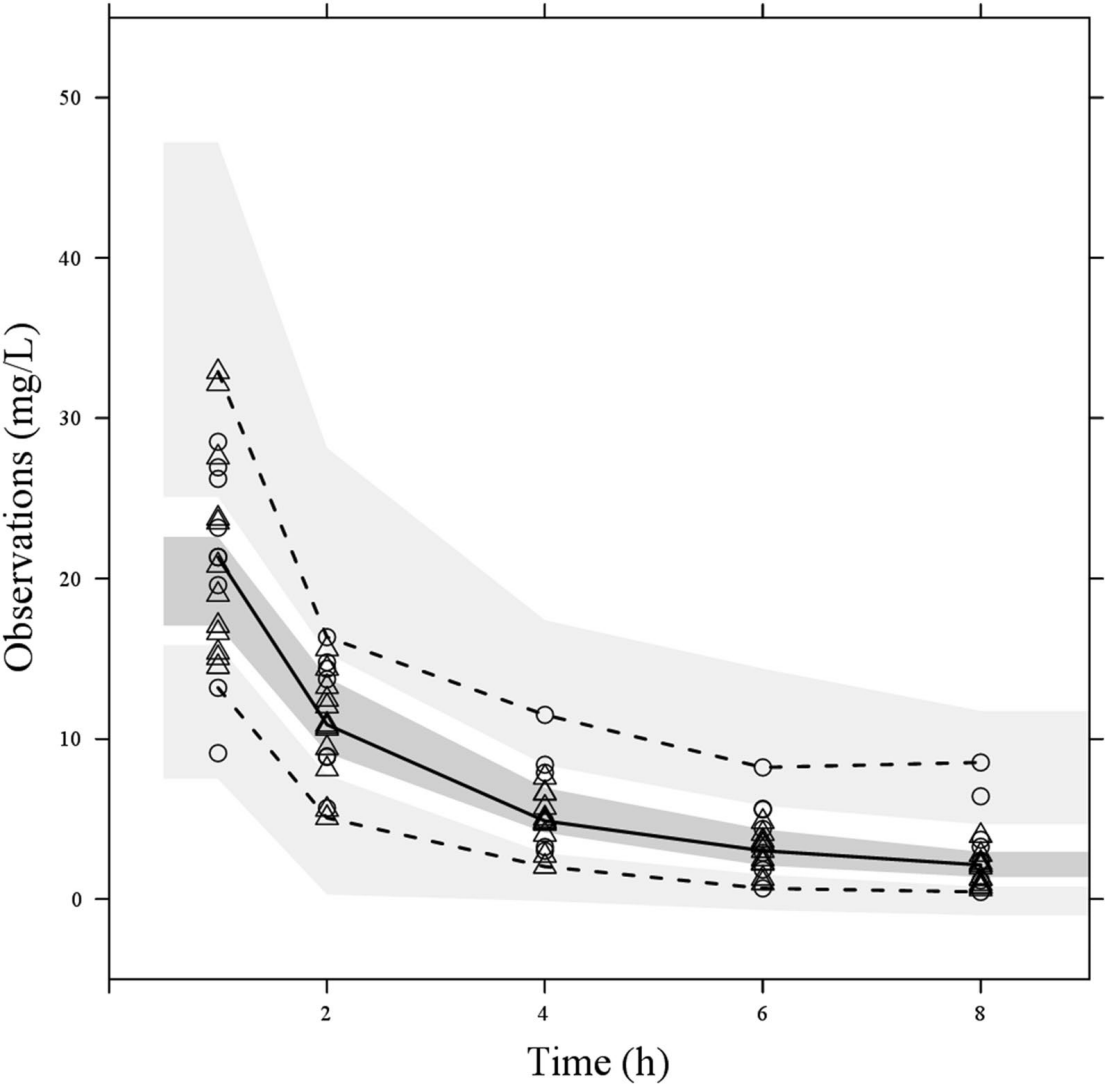
Visual predictive check for the final model. The open circles show observations for CRRT group and the open triangles for non-CRRT group. The solid line shows the fiftieth percentile of the observations, and the dashed lines show the fifth and ninety-fifth percentiles of the observations. The shaded areas show the 95% confidence intervals of the fifth, fiftieth, and ninety-fifth percentiles of the predictions.

The measured DRPM concentrations correlated well with the predicted concentrations both for population and individual predictions (Fig. 4). The conditional weighted residuals (CWRES) and individual weighted residuals (iWRES) distributed uniformly regardless of concentration and time. The above results suggested that the analyses of our model were valid. A comparison of each PPK parameter in the final model with each parameter obtained from 1000 bootstrap samples estimated no large error between the two, and the 95% confidence intervals were relatively small (Table 3). Visual predictive check was performed based on 1000 replicates. As shown in Fig. 5, almost all measured values were within the 95% confidence intervals estimated from our model. Thus, the reliability and stability of each PK parameter are proven and our model is valid.

**Table 3:** Results of population pharmacokinetic parameters for the final model and for bootstrap sampling.

Parameters	Base model		Population mean		Bootstrap (1000 replicates)	
	Estimates	Inter-individual variability (%)	Estimates	Inter-individual variability (%)	Median	95% CI
CL_body(non-CRRT)_ (L h^−1^)	3.89	17.0	3.65	7.3	3.59	2.63–4.24
CL_body(CRRT)_ ( L h^−1^)	1.83	29.4	2.49	22.2	2.49	1.58−4.11
V_1_ (L)	6.76	32.1	10.04	13.2	7.47	3.18−10.06
V_2_ (L)	8.54	16.6	8.13	24.2	8.91	6.36−11.38
Q ( L h^−1^)	4.62	9.4	3.53	12.7	4.59	3.60−5.52
Residual variability (μg mL^−1^)	0.016	42.3	0.70	36.5	0.02	0.01−0.03

*CL*_*body(non-CRRT)*_ clearance from body without continuous renal replacement therapy, *CL*_*body(CRRT)*_ clearance from body with continuous renal replacement therapy, *V*_*1*_ distribution volume of central compartment, *V*_*2*_ distribution volume of peripheral compartment, *Q* distribution clearance between the central and peripheral compartments, *RSE* relative standard error, *CI* confidence interval.

### Model simulation

Using our final model, Monte Carlo simulations were performed to evaluate the probability of target attainment (PTA) for targets of 40% and 100% of the time in which free drug concentrations exceed the MIC (40% fT > MIC and 100% fT > MIC, respectively) as well as 100% of the time in which free drug concentrations exceed 4 times the MIC (100% fT > MIC × 4). According to a previous report^30^, the following dosing regimens were simulated for the above three targets: 1-h intermittent infusion (InI), 4-h extended infusion (ExI), and continuous infusion (CI). Infusion interval was set at 8 h for InI and ExI. Simulated doses per infusion of DRPM were 250 mg, 500 mg, 1000 mg and 2000 mg for InI and Exl, but not for CI (for CI, the dose of DRPM was adjusted so that the daily dose was equal). The PTA was estimated for *Pseudomonas aeruginosa* (MIC ≤ 2) according to the European Committee on Antimicrobial Susceptibility Testing (EUCAST)^31^.

As shown in Table 4, PTA when administered by ExI and CI was higher than that when given by InI in all the simulations. For the target of 40% fT > MIC, all three infusion methods of all dosages achieved over 90% PTA regardless of renal function and CRRT.

**Table 4:** Results of simulation showing probability of target attainment by administering doripenem in different dosing regimens against *Pseudomonas aeruginosa* with MIC ≤ 2, under various conditions of renal function and continuous renal replacement therapy.

Renal function	Infusion method	CRRT	Dose/infusion (mg)	PTA (%) for		
				40%fT > MIC	100% fT > MIC	100% fT > MICx4
0 < Ccr ≤ 30	InI	On	2000	99.7	86.6	81.5
			1000	99.5	81.5	61.6
			500	99.0	73.7	39.9
			250	98.2	61.6	12.2
		Off	2000	100.0	97.5	94.5
			1000	100.0	96.4	91.0
			500	100.0	94.5	82.8
			250	99.0	91.0	66.7
	ExI	On	2000	100.0	100.0	100.0
			1000	100.0	100.0	99.4
			500	100.0	100.0	94.3
			250	100.0	99.4	58.8
		Off	2000	100.0	100.0	100.0
			1000	100.0	100.0	100.0
			500	100.0	100.0	99.8
			250	100.0	100.0	91.4
	CI	On	6000	100.0	100.0	100.0
			3000	100.0	100.0	99.5
			1500	100.0	100.0	95.5
			750	99.5	99.5	72.6
		Off	6000	100.0	100.0	100.0
			3000	100.0	100.0	100.0
			1500	100.0	100.0	99.9
			750	100.0	100.0	95.4
30 < Ccr ≤ 60	InI	On	2000	98.9	74.8	58.8
			1000	98.5	68.5	44.4
			500	97.8	58.8	24.1
			250	95.8	44.4	5.1
		Off	2000	99.8	87.3	75.9
			1000	99.6	82.9	63.9
			500	99.4	75.9	44.3
			250	99.0	63.9	20.6
	ExI	On	2000	100.0	100.0	99.8
			1000	100.0	100.0	98.4
			500	100.0	99.8	85.1
			250	99.8	98.4	35.1
		Off	2000	100.0	100.0	100.0
			1000	100.0	100.0	99.9
			500	100.0	100.0	92.5
			250	100.0	99.9	57.8
	CI	On	6000	100.0	100.0	99.8
			3000	100.0	100.0	98.5
			1500	99.8	99.8	88.0
			750	98.5	98.5	49.6
		Off	6000	100.0	100.0	100.0
			3000	100.0	100.0	99.9
			1500	100.0	100.0	96.9
			750	99.9	99.9	70.3
60 < Ccr ≤ 90	InI	Off	2000	99.4	75.9	59.4
			1000	98.9	69.3	44.5
			500	98.3	59.4	23.6
			250	96.8	44.5	7.6
	ExI	Off	2000	100.0	100.0	100.0
			1000	100.0	100.0	99.7
			500	100.0	100.0	86.4
			250	100.0	99.7	33.0
	CI	Off	6000	100.0	100.0	100.0
			3000	100.0	100.0	99.8
			1500	100.0	100.0	89.7
			750	99.8	99.8	45.5

*InI* intermittent infusion, *ExI* extended infusion, *CI* continuous infusion, *fT* > *MIC* time in which free drug concentrations exceed MIC, *PTA* probability of target attainment, *CRRT* continuous renal replacement therapy.

For the targets of 100% fT > MIC and 100% fT > MIC × 4, however, InI achieved over 90% PTA only in patients with renal function 0 < Ccr ≤ 30 not using CRRT, while all other renal function and CRRT conditions required ExI or CI to achieve 90% PTA. For patients using CRRT, ExI of a higher dosage was needed to achieve over 90% PTA for 100% fT > MIC × 4 versus 100% fT > MIC when renal function was 0 < Ccr ≤ 30 (500 mg vs. 250 mg or above) and 30 < Ccr ≤ 60 (1000 mg vs. 250 mg or above). For patients not using CRRT, the two dosages administered by ExI for the targets of 100% fT > MIC × 4 versus 100% fT > MIC were either not different (250 mg or above for both in 0 < Ccr ≤ 30) or higher for 100% fT > MIC × 4 (500 mg vs. 250 mg or above in 30 < Ccr ≤ 60; 1000 mg vs. 250 mg or above for 60 < Ccr ≤ 90). Note that a similar trend was observed for InI in patients with 0 < Ccr ≤ 30 not using CRRT (100% fT > MIC × 4 vs. 100% fT > MIC: 1000 mg vs. 250 mg or above).

When administered by CI, all dosages (750–6000 mg) achieved over 90% PTA for 100% fT > MIC and doses of 3000 mg or above achieved over 90% PTA for 100% fT > MIC × 4, for all conditions regardless of CRRT or renal function.

## Discussion

In our final PPK model for DRPM, CL_tot_ [CL_body(CRRT)_ and CL_body(non-CRRT)_] tends to be lower and V_2_ is higher compared to previous reports^6,8,11^. These differences probably arise because the subjects of previous studies included healthy individuals. PK of DRPM is expected to be greatly different between critically ill patients and healthy subjects, since a fluid retention tendency and augmented systemic inflammatory response are observed in the patients^32^.

The pathological conditions of patients in this study were diverse, including bacteremia, septic shock, infective endocarditis, pneumonia, intraperitoneal infection, urinary-tract infection, and poor infection control after surgery or transplantation. Previous reports on PPK models in patients undergoing CRRT focused on acute infections or sepsis^7,26^. Our PPK model was not developed targeting specific diseases and included patients using CRRT, which may more realistically reflect the critical clinical setting in which patients have diverse pathological conditions that could change rapidly. Thus this model may complement existing models, especially for disease conditions that change rapidly or for which PPK models have not been established.

In the final model, Ccr was a significant covariate for CL_body_. When CRRT is administered, serum creatinine is cleared via two routes: the kidney and CRRT. In the CRRT group, Ccr calculated from serum creatinine is considered inappropriate as an indicator of kidney function for the kidney alone, but appropriate as an indicator of overall renal function for the kidney combined with CRRT. Therefore, Ccr calculated from serum creatinine indicated renal function in different conditions depending on the presence or absence of CRRT. Hence, when used as a covariate in the model, Ccr has to be differentiated depending on the presence or absence of CRRT. That is why different median values of Ccr were used in the final models for the CRRT group and non-CRRT group. Although Ccr is not stable in patients with severe diseases, past reports^7,8,26^ have indicated that Ccr is a useful marker of renal function in the acute phase. By incorporating Ccr in CL_body_ into the base model, the inter-individual of CL_body_ in the final model has become smaller (non-CRRT group: 17.0–7.3%, CRRT group: 29.4–22.2%).

Roberts et al.^20^ reported that the contribution of CL_CRRT_ to CL_total_ was 30–37%. The renal function of the patients undergoing CRRT in this study showed some variations [Ccr; 57.3 ± 8.85 (mean ± SE)]. Therefore, these patients showed CL_total_ of 3.81 ± 0.31 (mean ± SE) and CL_CRRT_ of 1.31 ± 0.19, and the contribution of CL_CRRT_ to CL_total_ was 40.0 ± 3.92%. Our result of the contribution of CL_CRRT_ to CL_total_ was the same as past report, indicating the importance to consider drug removal by CRRT.

Achievement of the maximal bactericidal effect requires at least 40% fT > MIC^33^. However, it would be necessary to aim for more aggressive exposure of 100% fT > MIC or 100% fT > MIC × 4 in critically ill patients^34,35^. Hence, we simulated the above three targets for four dosages using three infusion methods (InI, ExI and CI) for each condition of CRRT and renal function. As shown in Table 4, the PTA of InI was inferior to that of ExI and CI, and more than 90% PTA for 100% fT > MIC and 100% fT > MIC × 4 could not be achieved in most conditions. For non-critically ill patients, if the drug exposure target was set at 40% fT > MIC, efficacy against bacteria with MIC 2 or less can be expected by administering 250 mg or more by InI every 8 h, regardless of renal function or CRRT. On the other hand, for critically ill patients not undergoing CRRT, if the drug exposure target was set at 100% fT > MIC, DRPM should be administered by ExI or CI, except for patients with 0 < Ccr ≤ 30 not undergoing CRRT. Using CI, dosages of 3000 mg or above achieved over 90% PTA for even 100% fT > MIC × 4 for all conditions regardless of CRRT or renal function. However, these dosage regimens are expected to be effective only based on simulations. A recent systematic review and meta-analysis of critically ill patients indicated no significant difference in effectiveness between CI and InI^36^. On the other hand, according to a previous meta-analysis comparing ExI and InI, ExI had a higher clinical success rate and lower mortality than InI^37^. Considering the possible toxic effect of CI, we recommend that DRPM should be administered by ExI but not by CI to achieve over 90% PTA for 100% fT > MIC and 100% fT > MIC × 4.

Our report had two limitations. First, we were not able to measure DRPM concentrations in the filtrate or plasma concentrations during blood removal and blood returning. Therefore, we were unable to calculate the CL_CRRT_ from measured data. However, DRPM is removed by simple hemodiafiltration process, since it is unnecessary to consider the adsorption process of DRPM at the cellulose triacetate membrane^27^. Second, this study was conducted in a single facility, and the numbers of subjects and samples were not sufficient. A multicenter joint study is needed to validate the model.

In conclusion, PPK analysis was performed in patients who required systemic management at the ICU and received DRPM for severe infections. Because this population included patients undergoing CRRT, we constructed a model that incorporate the DRPM drug excretion pathway by CRRT. Using this model, Ccr was selected as a significant covariate for CL_body_. The contribution rate of CL_CRRT_ to CL_total_ was 40 ± 3.92%. Furthermore, the results of Monte Carlo simulation show a possibility that DRPM clearance may be significantly different depending on the presence or absence of CRRT, which may impact the therapeutic effect. The present findings thus suggest that drug removal by CRRT may be important. From the above, our novel model is a useful tool for deciding administration of DRPM and may contribute greatly to further proper use of DRPM in patients requiring intensive care.

## Supplementary Information

Supplementary Information.
