# Supplementary material for: Population pharmacokinetic analysis of doripenem for Japanese patients in intensive care unit
Source: Sci Rep. 2020 Dec 17;10:22148. doi: 10.1038/s41598-020-79076-6 (PMC7747597; doi:10.1038/s41598-020-79076-6)
Supplement: Supplementary file 1 — Supplementary Information. [file 41598_2020_79076_MOESM1_ESM.docx]

**Supplemental Data**

**Population pharmacokinetic analysis of doripenem for Japanese patients in intensive care unit**

Ko Nonoshita^1*^, Yosuke Suzuki^1^, Ryota Tanaka^1^, Tetsuya Kaneko^1^, Yoshifumi Ohchi^2^, Yuhki Sato^1^, Norihisa Yasuda^2^, Koji Goto^2^, Takaaki Kitano^2^, Hiroki Itoh^1^

^1^Department of Clinical Pharmacy, Oita University Hospital, Hasama-machi, Oita, Japan; ^2^Department of Anesthesiology and Intensive Care, Oita University Faculty of Medicine, Hasama-machi, Oita, Japan

**Contents**

Table S1 Demographics and relevant clinical data of all patients.

Figure S1 Histogram of CL_body(non-CRRT)_ from bootstrap sampling.

Figure S2 Histogram of CL_body(CRRT)_ from bootstrap sampling.

Figure S3 Histogram of V_1_ from bootstrap sampling.

Figure S4 Histogram of V_2_ from bootstrap sampling.

Figure S5 Histogram of Q from bootstrap sampling.

**Table S1 Demographics** **and relevant clinical data of all patients.**

| ID | Age | Sex  (M/F) | Ht  (cm) | BW  (kg) | Dose  (g) | Ccr  (mL/min) | APACHE II | SOFA | Specimen | Detected bacteria | Treatment target |
| --- | --- | --- | --- | --- | --- | --- | --- | --- | --- | --- | --- |
| 1 | 29 | F | 160 | 48.5 | 0.5 | 105.9 | 11 | 9 | Endotracheal sputum | *A*. baumannii, *Pseudomonas* spp,  *H*. influenza | Suspected pneumonia |
| 2 | 73 | M | 173.3 | 71.6 | 0.5 | 62.3 | 19 | 8 | Endotracheal sputum | *E*. cloacae | Postoperative infection |
| 3 | 59 | M | 164.6 | 60.2 | 0.5 | 109.2 | 20 | 9 | Endotracheal sputum | Undetected | Sepsis, Pneumonia |
| 4 | 47 | M | 178 | 92.4 | 0.5 | 59.4 | 14 | 6 | Blood | Undetected | Postoperative infection |
| 5 | 80 | M | 157.2 | 66.8 | 0.5 | 64.7 | 15 | 6 | Endotracheal sputum | Undetected | Suspected pneumonia |
| 6 | 67 | M | 165 | 54.0 | 0.5 | 55.9 | 15 | 6 | Endotracheal sputum | *P*. aeruginosa | Pneumonia |
| 7 | 80 | F | 158 | 61.5 | 0.5 | 62.2 | 14 | 5 | Endotracheal sputum | *E*. aerogenes | Suspected pneumonia |
| 8 | 61 | M | 174.5 | 68.4 | 0.25 | 20.7 | 23 | 10 | Purulent discharge | *E*. avium, *Bacteroides* spp | Sepsis, Intraperitoneal infection |
| 9 | 33 | M | 145 | 28.8 | 0.5 | 93.0 | 11 | 6 | Blood | *E*. faecalis | Septic shock, Urinary-tract infection |
| 10 | 79 | M | 156 | 49.9 | 0.5 | 50.3 | 19 | 8 | Blood | *S*. epidermidis,  *Corynebacterium* spp | Septic shock, Intraperitoneal infection |
| 11 | 58 | M | 172.1 | 63.0 | 0.5 | 97.0 | 12 | 7 | Blood | Undetected | Postoperative infection |
| 12 | 81 | F | 140 | 46.4 | 0.5 | 68.8 | 17 | 7 | Blood | Undetected | Septic shock, Ileus postoperative enteritis |
| 13 | 56 | M | 167.5 | 60.0 | 0.5 | 155.6 | 24 | 6 | Blood | *S*. aureus | Sepsis, Purulent discitis |
| 14 | 86 | M | 165 | 59.0 | 0.5 | 50.3 | 17 | 6 | Endotracheal sputum | Undetected | Pneumonia |
| 15 | 60 | M | 164 | 70.0 | 0.5 | 54.4 | 8 | 7 | Endotracheal sputum | *E*. cloacae | Postoperative infection |
| 16 | 42 | M | 171 | 57.0 | 0.5 | 32.1 | 24 | 5 | Tissues  Endotracheal sputum | *Streptococcus* spp *E*. coli (ESBLs) | Sepsis, Infective endocarditis |
| 17 | 15 | M | 170 | 60.9 | 0.5 | 121.5 | 14 | 6 | Endotracheal sputum | Undetected | Unknown fever |
| 18 | 71 | M | 162 | 50.0 | 0.5 | 58.4 | 8 | 3 | Endotracheal sputum | Undetected | Postoperative infection |
| 19 | 73 | M | 165 | 60.7 | 0.5 | 25.2 | 21 | 12 | Endotracheal sputum | *S*. epidermidis | Sepsis, Pneumonia |
| 20 | 65 | M | 173 | 87.1 | 0.5 | 52.8 | 26 | 14 | Blood | Undetected | Sepsis, Postoperative infection |
| 21 | 83 | M | 167.5 | 75.0 | 0.5 | 28.5 | 37 | 12 | Endotracheal sputum | *M*. catarrhalis, *R*. mucilaginosa | Septic shock, Pneumonia |

Ht, height; BW, body weight; Ccr, creatinine clearance estimated by Cockcroft-Gault equation; APACHEII, acute physiology and chronic health evaluation II; SOFA, sequential organ failure assessment


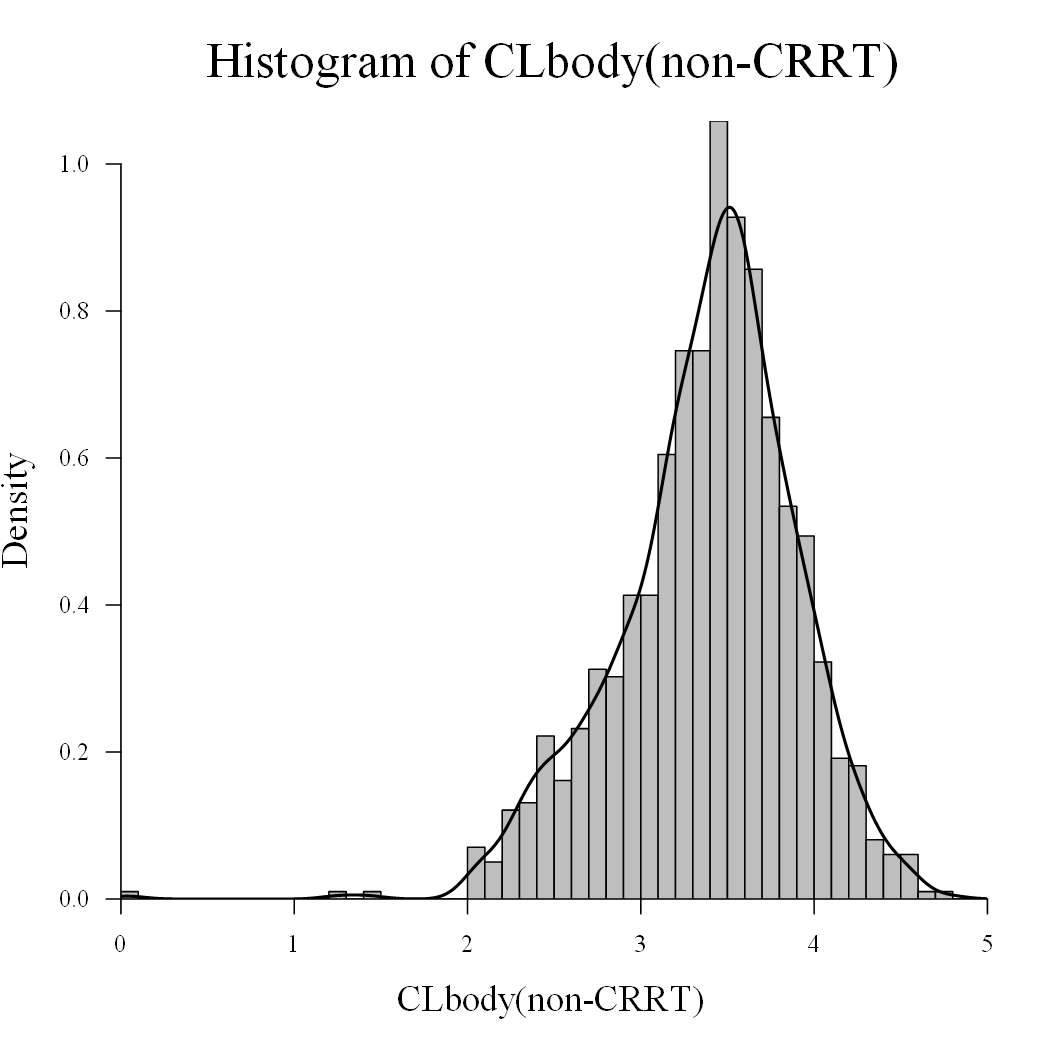


**Figure S1 Histogram of CL_body(non-CRRT)_ from bootstrap sampling.**


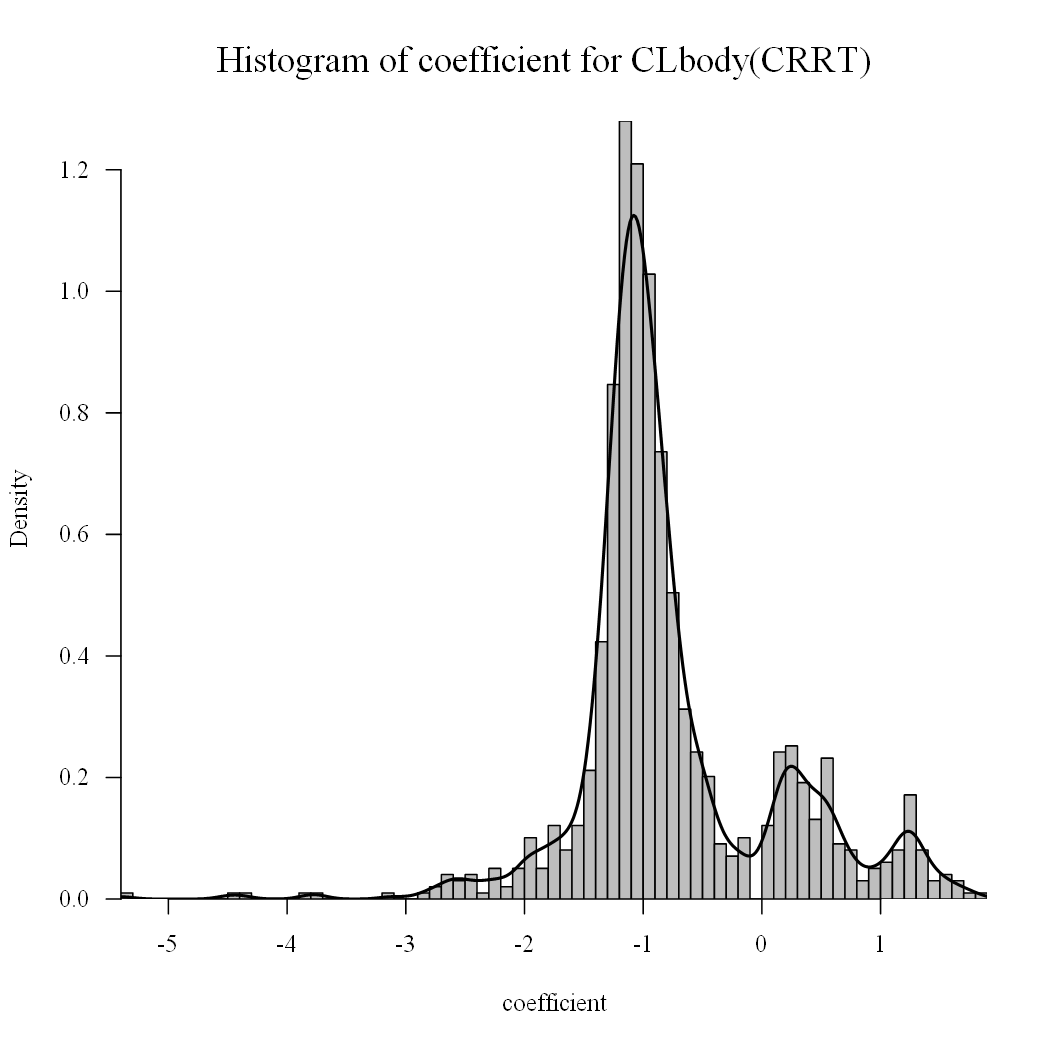


**Figure S2 Histogram of CL_body(CRRT)_ from bootstrap sampling.**


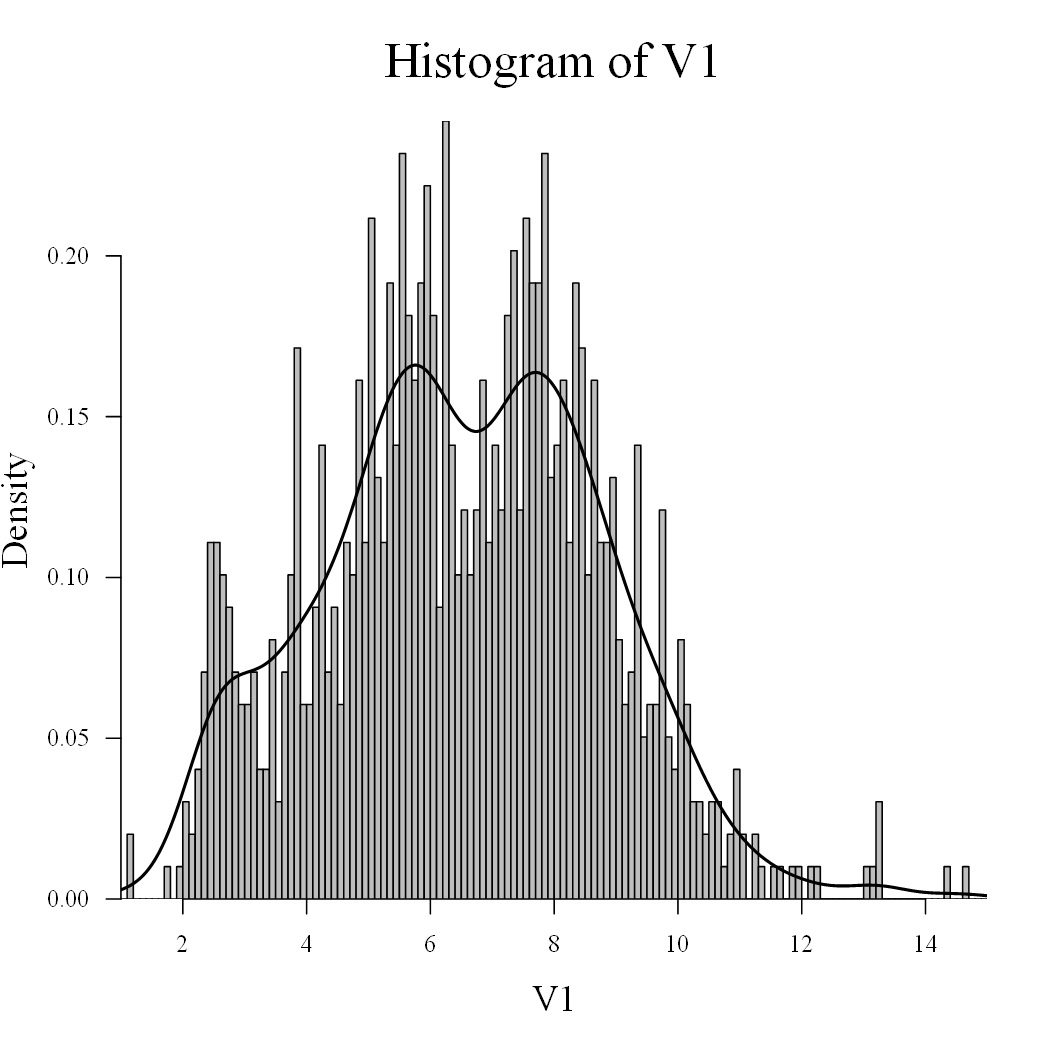


**Figure S3 Histogram of V_1_ from bootstrap sampling.**


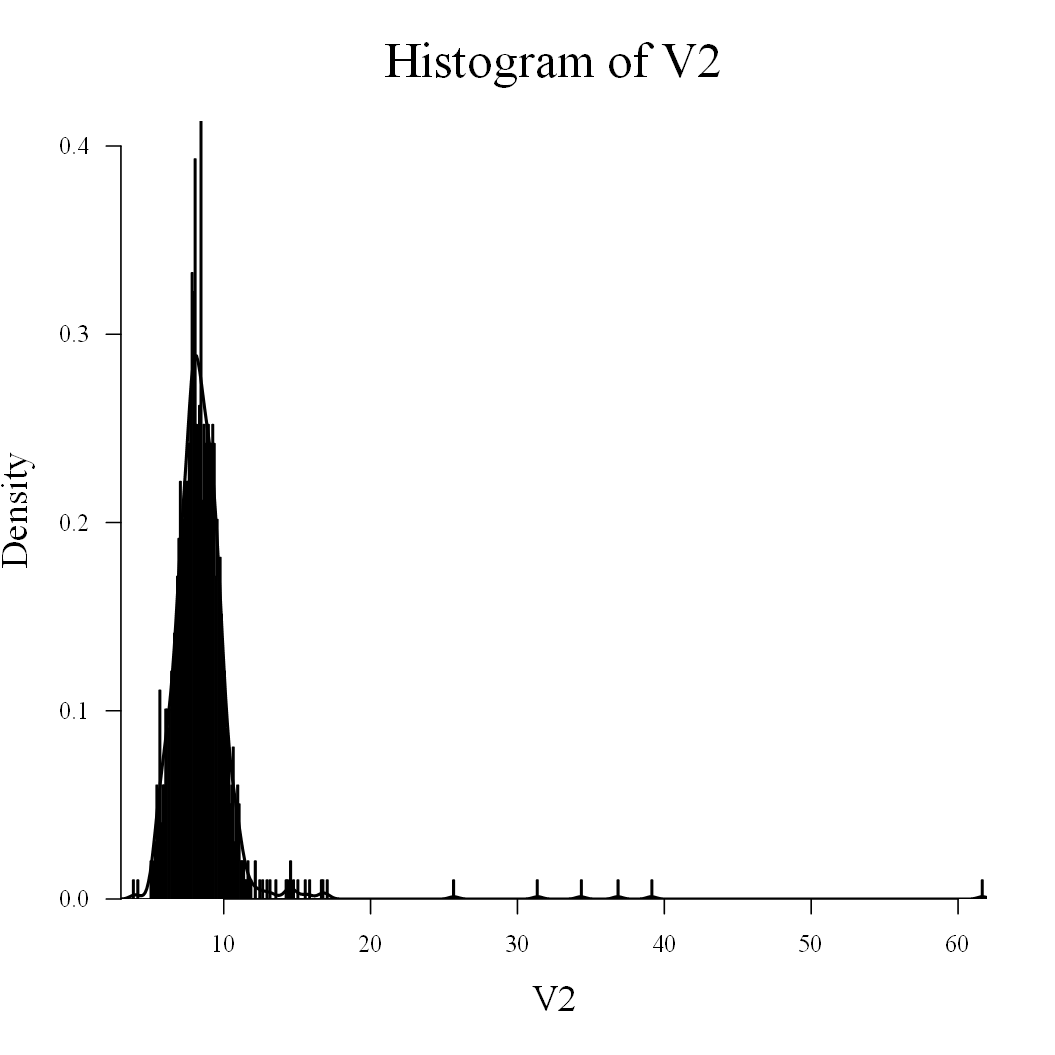


**Figure S4 Histogram of V_2_ from bootstrap sampling.**


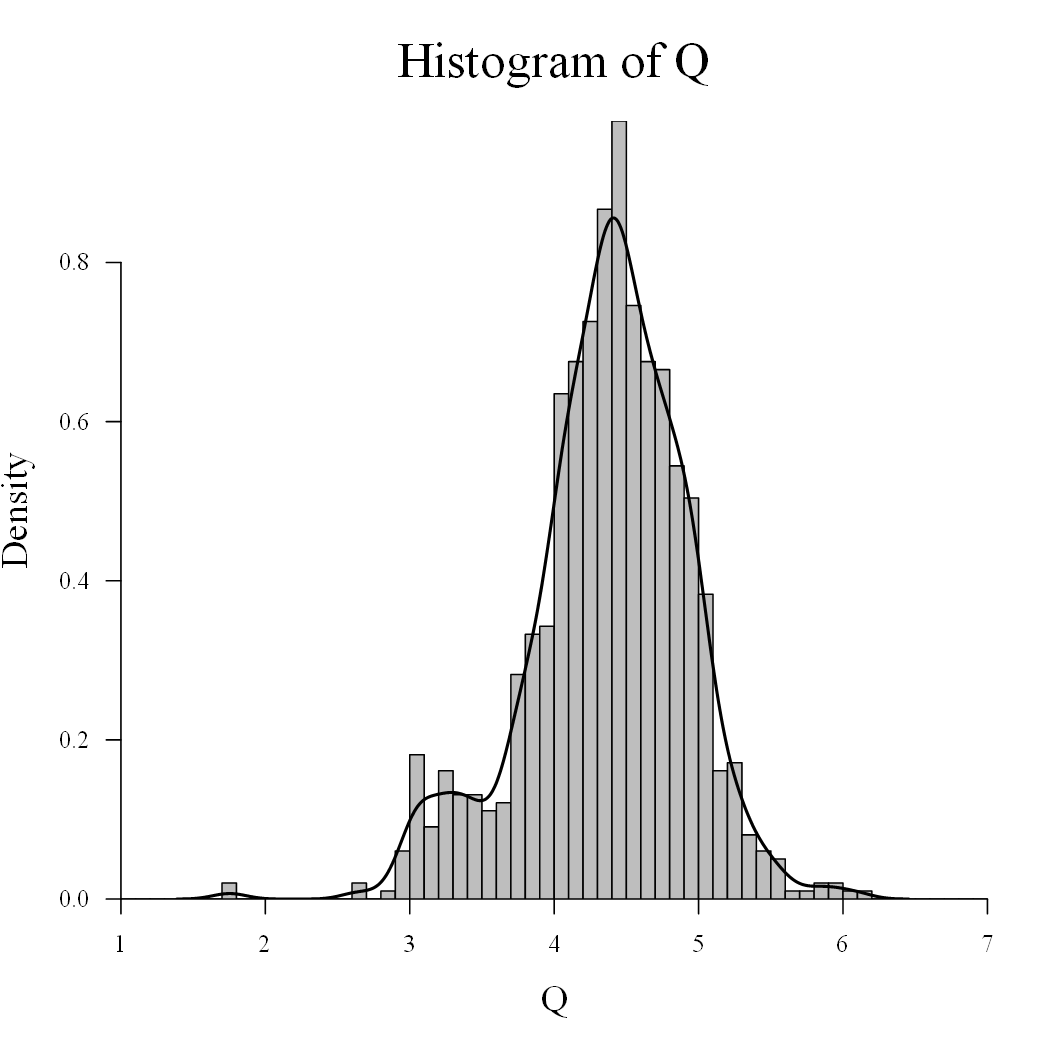


**Figure S5 Histogram of Q from bootstrap sampling.**
